# Splenic tuberculosis in an immunocompetent patient can be managed conservatively: a case report

**DOI:** 10.1093/gastro/gov058

**Published:** 2015-11-13

**Authors:** Ashok Kumar, V K Kapoor, Anu Behari, Sandeep Verma

**Affiliations:** Department of Surgical Gastroenterology, Sanjay Gandhi Postgraduate Institute of Medical Science, Lucknow, India

**Keywords:** splenic tuberculosis, immunocompetence, splenectomy

## Abstract

Tuberculosis is a significant health problem in developing countries. Splenic tuberculosis usually occurs as a part of miliary tuberculosis, and ranks third after lung and liver involvement, respectively. Splenic involvement is more common in immunocompromised patients and is very rarely found in immunocompetent patients. Here we report a case of splenic tuberculosis in an immunocompetent patient, which was managed successfully with conservative treatment.

## Introduction

Tuberculosis is a significant health problem in developing countries and extrapulmonary involvement is an important disease entity in India. The involvement of abdominal organs is seen only in 11% of extrapulmonary cases, the spleen being, after the lungs (100%) and liver (82%), the third most common organ involved in the setting of miliary tuberculosis [1–4]. Though mostly reported in immunocompromised patients, it has on rare occasions been seen to occur also in immunocompetent patients. The exclusion of splenic tuberculosis is necessary as a differential diagnosis of fever of unknown origin, especially in areas in which the disease is endemic. There are also some reports of spontaneous splenic abscess, causing acute abdominal pain and clinical hypersplenism with bleeding tendencies. We report a case of isolated splenic tuberculosis in an immunocompetent patient.

## Case report

A 51-year-old male presented himself at our institution with a 3-month history of high-grade fever and left-side, upper abdominal pain, unrelated to radiation or food. The abdominal pain was aggravated by lying down on the left side and was not associated with nausea, vomiting, melena, or change in bowel habit or urinary symptoms. He had no history of cough, breathlessness, any addiction, or previous surgery. On physical examination, the patient was well orientated, well nourished and with good performance status. Abdominal examination revealed a moderate, tender splenomegaly, palpable just below the costal margin, with smooth surface and firm consistency. The abdomen was non-distended, with no free fluid. Haemogram analyses revealed that haemoglobin was 62 g/L, total lymphocyte count 18.0, and Differential leukocyte count (DLC) 81/12/5/2. Liver function tests revealed that serum bilirubin was 28 mg/L (direct: 7 mg/L), glutamic-oxalacetic transaminase (SGOT) 90 IU/L, glutamic-pyruvic transaminase (SGPT) 143 IU/L, alkaline phosphatase 94.6 IU/L. ELISA (enzyme linked immunosorbent assay) for HIV (Human immunodeficiency virus) was negative (no antibody).

**Figure 1.**
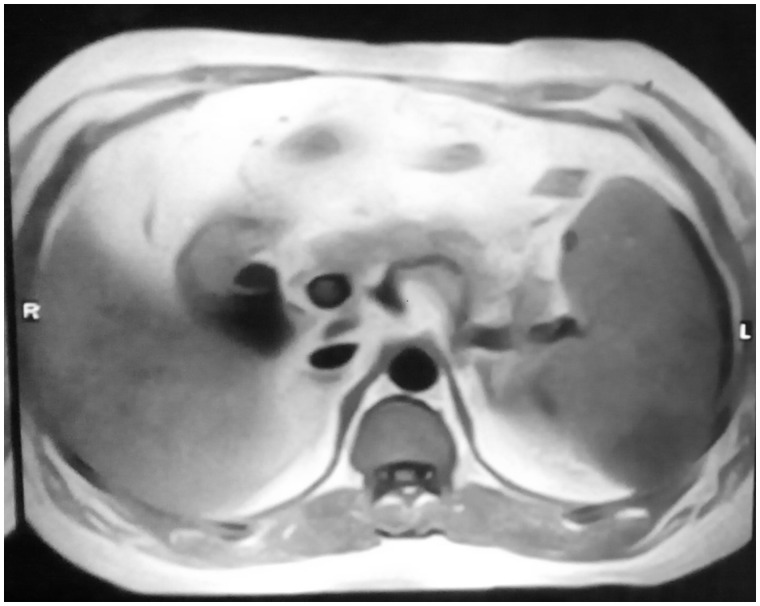
Magnetic resonance cholangiopancreatography showing a hyperintense area within the spleen on T2-weighted imaging, with splenomegaly

**Figure 2.**
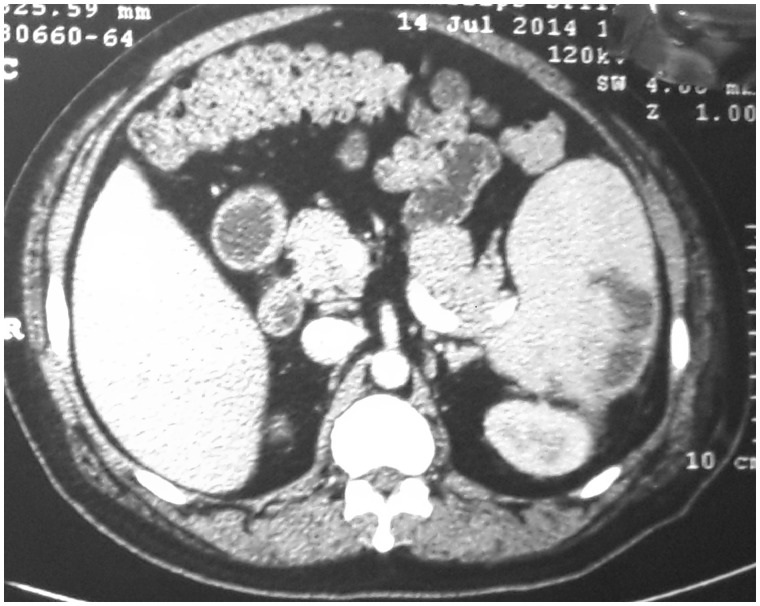
Contrast-enhanced spiral CT scan showing splenomegaly with multiple hypodense cystic lesions with ill-defined margins

Abdominal ultrasound examination revealed a normal liver, a single large calculus in the gallbladder, with normal wall thickness and a normal common bile duct (CBD), portal vein and pancreas, and splenic size was 15.7 cm. Ascites and enlarged lymph nodes were absent. Magnetic resonance imaging (MRI) confirmed the presence of the gall bladder calculus and normal CBD, and showed a hyperintense area within the spleen on T2-weighted imaging, with splenomegaly (Figure 1). Contrast-enhanced computed tomography (CECT) of the abdomen revealed a hypodense lesion 120 x 50 mm in size in the spleen, with splenomegaly; impression was splenic abscess with minimal left-sided pleural effusion (Figure 2). A diagnosis of splenic tuberculosis was made by the findings of a CT-guided fine needle aspiration cytology (FNAC).

In the initial phase, the patient was given anti-tubercular treatment with a four-drug regimen of isoniazid, rifampcin, ethambutol and pyrazinamide for two months, followed by isoniazid and rifampcin for the next four. He responded well and the high-grade fever and left-sided pain subsided. CT scans at 3 and 6 months showed a resolving lesion in the spleen. On 8 months follow-up, the patient is doing well, has regained weight and a good appetite and has had no recurrence to date.

## Discussion

Splenic abscess is a rare disease entity, autopsy studies reporting an incidence of only 0.2–0.7%. If these patients are left untreated, mortality is very high [5, 6]. Splenic involvement usually occurs as a part of miliary tuberculosis; however isolated involvement is also reported [7–9]. Only eight cases with primary tuberculous splenic abscess have been described in the literature; seven cases were diagnosed on the basis of histopathological findings on splenic biopsy, splenectomy, splenic abscess drainage and exploratory laparotomy [1, 3, 7, 8, 10]. Only one case was diagnosed on the basis of radiological findings on CT scan of the abdomen, and biochemical marker interferon gamma assay and adenosine deaminase [11].

A presentation of fever with chills and left upper abdominal pain—as seen in our case—is usually not seen in splenic tuberculosis. Ultrasonography is a non-invasive, cost-effective tool for diagnosis and for assessing therapeutic response in splenic tuberculosis. The most common findings are single or multiple hypoechoic focal lesions, splenic abscesses, calcification and isolated splenomegaly [12]. According to pathomorphological classification, there are five types of finding on ultrasound of splenic tuberculosis: miliary, nodular, tubercular splenic abscess, calcific, and mixed type. CT scan of the abdomen is the most reliable imaging investigation for tubercular splenic abscess, which is seen as a low-density mass with peripheral enhancement on intravenous contrast infusion. CT also delineates the exact location of the lesion and helps in planning therapeutic strategies, such as percutaneous drainage [13]; however, definite diagnosis can only be made on cytology or histopathology: typical findings are caseating granuloma of epithiloid and Langhan cells [9]. Real-time PCR is a very useful investigation for directly detecting the tubercle bacilli in clinical specimens. Laparoscopy is a very useful, less-invasive method of obtaining any form of splenic biopsy and avoiding splenectomy in patients who are reluctant to undergo surgery [14].

In most reports of splenic tuberculosis, patients have been treated by percutaneous aspiration or splenectomy followed by anti-tubercular treatment, or anti-tubercular treatment alone. In all these cases, the disappearance of splenic hypodensity and complete response to medication was another indicator to confirm the diagnosis of tuberculous splenic abscess. In our patient—who presented with high-grade fever with abdominal pain—there was no personal or family history suggestive of pulmonary tuberculosis, and no indication or sign of the involvement of other organs. Our patient was diagnosed on the basis of the findings of a CT scan and MRI of the abdomen, and the diagnosis was confirmed by CT-guided FNAC. The patient recovered completely on a 4-drug regimen of anti-tubercular treatment and splenectomy was avoided. The treatment of splenic tuberculosis is the same as proposed for pulmonary tuberculosis: standard anti-tubercular treatment should be given in combination for at least 6 months and the entire course should be completed, whether or not an operation is performed. Splenectomy can be avoided in cases where good response is seen. Splenectomy is only indicated in patients who fail to respond to anti-tubercular medication.

In summary, patient selection for a specific intervention modality is very important in improving prognosis. Early detection and less-invasive management with combination of anti-tubercular drugs may be successful in immunocompetent splenic tuberculosis patients. Splenectomy can be avoided in these patients and may be reserved for patients with splenic rupture and in cases of failed anti-tubercular treatment.
